# Clinical Application of Multi-Index Combined Risk Assessment in Early Pregnancy for Screening of Preeclampsia

**DOI:** 10.1155/2022/5089442

**Published:** 2022-09-27

**Authors:** Xiaohong Xu, Guoxiu Yan, Jijun Liu, Xuelei Li, Bin Zhang, Xianglian Meng, Hongbo Chen, Baoliang Han, Kun Shao, Xuefen Zhao, Jing Liu, Yan Yan

**Affiliations:** ^1^Department of Clinical Laboratory, Anhui Provincial Maternity and Child Health Hospital, Hefei, China; ^2^Department of Ultrasound, Anhui Provincial Maternity and Child Health Hospital, Hefei, China; ^3^Department of Science and Education, Anhui Provincial Maternity and Child Health Hospital, Hefei, China; ^4^Department of Obstetrics and Gynecology, Anhui Provincial Maternity and Child Health Hospital, Hefei, China; ^5^Department of Clinical Laboratory, Lu'an City Jin'an District Maternal and Child Health Care Hospital, Lu'an, China; ^6^Department of Clinical Laboratory, Fuyang Women and Children's Hospital, Fuyang, China; ^7^Department of Obstetrics and Gynecology, Lu'an City Maternal and Child Health Care Hospital, Lu'an, China; ^8^Prenatal Screening Center, Bozhou Women and Children's Health Hospital, Bozhou, China; ^9^Ministry of Women's Health, Chuzhou Maternal and Child Care Family Planning Service Center, Chuzhou, China

## Abstract

**Objective:**

To explore the predictive value of single-index screening or multi-index combined screening for preeclampsia.

**Methods:**

From January 1, 2019, to December 31, 2021, pregnant women with a singleton pregnancy who had been regularly checked in each center since the first trimester (between 11 and 14 weeks of gestation) were retrieved from multiple participating centers. The risk calculation software LifeCycle 7.0 was used to calculate the risk values before 32 weeks, 34 weeks, and 37 weeks of gestation, and through a receiver operating characteristic (ROC) curve analysis, the predictive values of pregnancy-associated protein A (PAPP-A), the placental growth factor (PLGF), the mean arterial pressure (MAP), the uterine artery pulsatility index (UTPI), or a combined multi-index were calculated for preeclampsia.

**Results:**

Finally, 22 pregnant women developed preeclampsia, and the area under the ROC curve of the PAPP-A + PLGF + MAP + UTPI combined screening program was greater than that of other screening programs before 37 weeks of gestation (AUC = 0.975, 0.946, or 0.840 for <32 weeks, <34 weeks, or <37 weeks, respectively). At 32 weeks, the Youden index was at its maximum.

**Conclusion:**

PAPP-A + PLGF + MAP + UTPI combined screening is the optimal screening mode for preeclampsia screening before 37 weeks of gestation, and the combined prediction using multiple indicators in early pregnancy is more suitable for predicting the risk of early-onset preeclampsia.

## 1. Introduction

Preeclampsia refers to elevated blood pressure and proteinuria after 20 weeks of gestation, which may be accompanied by symptoms such as damage to multiple organs or systems of the whole body. It is an important cause of maternal death, fetal growth restriction, and premature fetal birth. Studies have shown that taking aspirin before 16 weeks of gestation in high-risk women can significantly reduce the risk of preeclampsia [1]. Therefore, monitoring the occurrence of preeclampsia in the first trimester has significant social significance for ensuring maternal and fetal safety and reducing the incidence of birth defects. There are many screening programs for preeclampsia, and traditional screening is based on maternal factors with low reliability. In order to evaluate the reliability of preeclampsia screening before 37 weeks of gestation, this study combined pregnancy-associated protein A (PAPP-A), placental growth factor (PLGF), mean arterial pressure (MAP), and uterine artery pulsatility index (UTPI) in the first and second trimesters of pregnancy. Multiple index screening methods are used to predict preeclampsia, and each screening index alone or the combined indexes were evaluated for preeclampsia screening. This study aims to provide a basis for the establishment of screening programs for preeclampsia in early pregnancy.

## 2. Materials and Methods

### 2.1. General Information

This study was conducted by Anhui Maternal and Child Health Hospital in collaboration with Lu'an Jin'an District Maternal and Child Health Hospital, Fuyang Women and Children's Hospital, Lu'an Maternal and Child Health Hospital, Bozhou Maternal and Child Health Hospital, and Chuzhou Maternal and Child Health and Family Planning Service Center. The study protocol was approved by the ethical committee of all participating hospitals. In this collaborative multicenter study on serological screening for preeclampsia, pregnant women with singleton pregnancies who started regular obstetric examinations at various hospitals from January 1, 2019, to December 31, 2021, since the first trimester (11–14 weeks of gestation) were retrieved. Inclusion criteria were: pregnant women with a singleton pregnancy who received early-trimester serological screening for Down syndrome at various hospitals at 11–14 weeks of gestation. Exclusion criteria were: (1) multiple pregnancies; (2) maternal complications that may affect the occurrence of gestational hypertension symptoms and comorbidities; (3) fetal malformation and chromosomal disease; and (4) miscarriage, fetal death, or termination of pregnancy before 28 weeks of gestation. A total of 9000 pregnant women were enrolled, of which 2862 were excluded for the following reasons: hypertension not related to preeclampsia (*n* = 747), immune diseases (*n* = 459), gestational diabetes mellitus (*n* = 253), miscarriage, termination of pregnancy (*n* = 615), and lost to follow-up (*n* = 788). Finally, 6138 pregnant women were included in the study, followed up until 37 weeks of gestation, and all pregnant women signed the informed consent form after consultation.

### 2.2. Observation Indicators and Evaluation Criteria

Fasting venous blood samples (2-3 ml) of the pregnant women who participated were collected. The serological indexes PAPP-A and PLGF were detected using the Auto DELFIA 1235 automatic time-resolved fluorescence immunoassay analyzer (PerkinElmer, USA). The placental growth factor determination kit and pregnancy-related protein A detection kit were used according to the manufacturer's manual [2].

Auxiliary diagnostic test indicators included: (1) the MAP was measured by an electronic sphygmomanometer, and the systolic and diastolic blood pressures were recorded (the difference between the diastolic blood pressures on both sides was less than 6 mm·Hg, and the difference in systolic blood pressure was less than 10 mm·Hg), and the average was taken. MAP = (systolic blood pressure + diastolic blood pressure × 2)/3; and (2) UTPI was checked by Doppler ultrasonography. The blood flow index of bilateral uterine arteries was detected at the point where the branch of the internal iliac artery crosses the uterine artery above the external iliac blood vessel, and each pregnant woman's bilateral uterine artery blood flow index was measured. The uterine artery Doppler blood flow spectrum was performed three times, and five complete, clear, consistent, and stable blood flow spectra were obtained each time. Finally, the average value of bilateral uterine artery blood flow, UTPI, was used for risk calculation.

Preeclampsia diagnostic criteria: systolic blood pressure ≥140 mm·Hg and/or diastolic blood pressure ≥90 mm·Hg after 20 weeks of gestation, accompanied by any of the following: urine protein quantitative >0.3 g/24 h, or random urine protein (+); no proteinuria but with any of the following organ or system involvement: eg., thrombocytopenia, hepatic or renal impairment, pulmonary edema, new-onset central nervous system abnormalities, or visual disturbances; preeclampsia with severe manifestations. In this study, those who met the above criteria were regarded as the preeclampsia group, and those who did not meet the above criteria were regarded as the control group.

### 2.3. Statistical Methods

SPSS 25.0 (SPSS, Chicago, IL, USA) was used for data processing, and the measurement data conforming to the normal distribution were expressed as the mean ± standard deviation ($\overset{–}{x}$ ***±*** *s*); otherwise, the nonparametric test was used, and the median and upper and lower quartiles were expressed. The values of PAPP-A, PLGF, MAP, and UTPI were analyzed using the LifeCycle7.0 risk assessment software developed by PerkinElmer (USA) to assess the risk of preeclampsia [3]. The effectiveness of each screening program in predicting preeclampsia was analyzed by the area under the curve (AUC) in the ROC curve, sensitivity, specificity, and the Youden index. The application value of the combined prediction scheme of PAPP-A, PLGF, MAP, and UTPI in predicting preeclampsia was analyzed, and the statistical difference was based on a 2-sided*P* < 0.05.

## 3. Results

### 3.1. Comparison of Basic Information of Two Groups of Pregnant Women

Among the 6138 pregnant women included in this study, there were 22 pregnant women in the preeclampsia group and 6116 pregnant women in the control group. The average expected delivery age of pregnant women was (27.85 ± 3.72) years old in the preeclampsia group and (28.10 ± 3.54) years old in the control group. The average weight of pregnant women was (60.27 ± 9.18) kg in the preeclampsia group and (56.93 ± 9.21) kg in the control group. The average height of pregnant women was (160.18 ± 4.85) cm in the preeclampsia group and (160.69 ± 4.96) cm in the control group. There was no statistical difference in prenatal age, weight, or height between the preeclampsia group and the control group (*P* > 0.05), as shown in Table 1.

The mean values of the multiple of the median (MoM) of each index of pregnant women in the control group and the preeclampsia group were compared. The Kolmogorov-Smirnov test (KS) normality test showed that the MoM values of MAP and PAPP-A were normally distributed. The mean value of PAPP-A MoM value (1.14 ± 0.12) in the preeclampsia group was higher than that of the control group (1.01 ± 0.11, *P* < 0.01), and the mean value of PAPP-A MoM value of pregnant women in the preeclampsia group (0.68 ± 3.60) was lower than that of the control group (1.13 ± 0.69, *P* < 0.01). Significant differences. The MoM values of PLGF and UTPI showed a nonnormal distribution. A nonparametric test was used. The average value of PLGF MoM in the preeclampsia group (0.38) was lower than that in the control group (0.77, *P* < 0.011), and the UTPI MoM value of the preeclampsia group (0.92) was higher than that of the control group (0.80, *P*=0.018).

### 3.2. Analysis of the Predictive Value of Different Screening Programs for Preeclampsia

The predictive values for the risk of preeclampsia using PAPP-A, PLGF, MAP, and UTPI programs (PAPP-A + PLGF + MAP + UTPI, PAPP-A + PLGF + MAP, PAPP-A + PLGF, PLGF + MAP, PAPP- A) before 32 weeks, 34 weeks, or 37 weeks of gestation were analyzed by the ROC curve.

**Figure 1:**
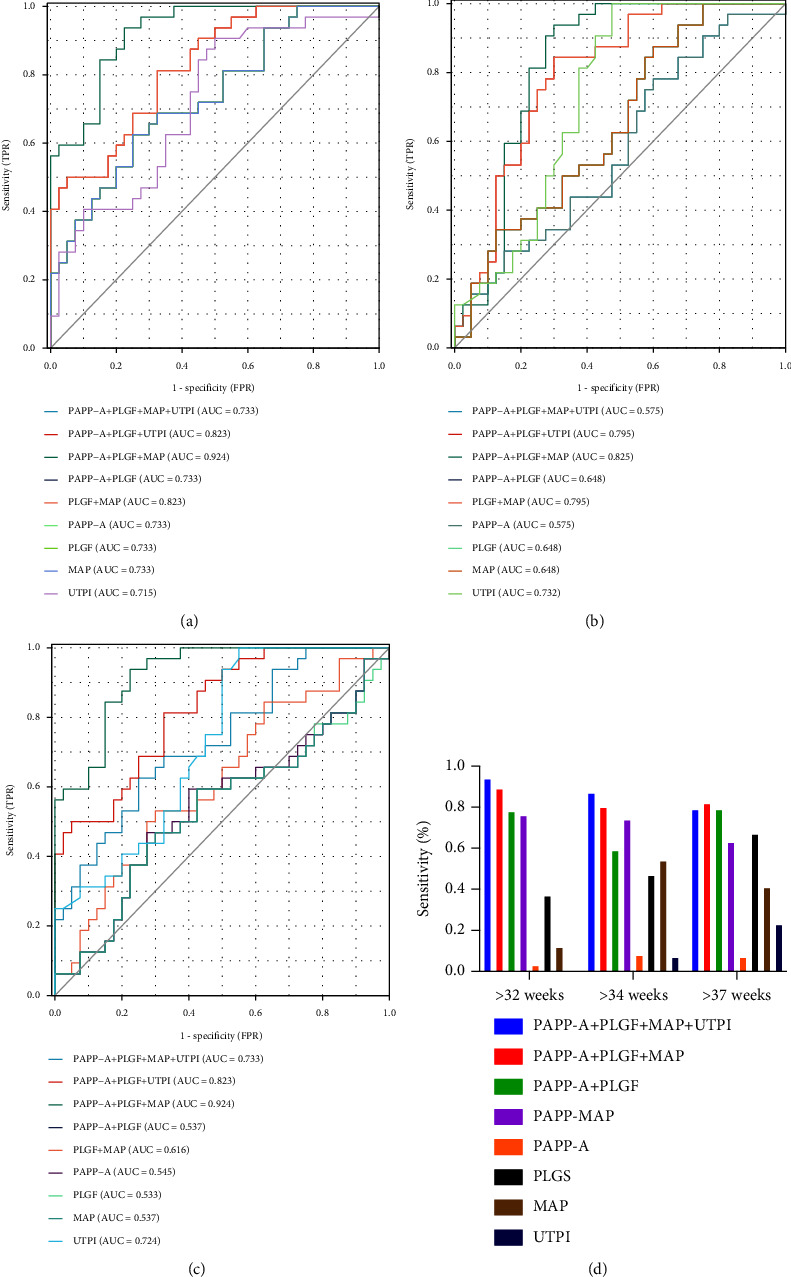
The ROC curves of different screening programs for predicting the risk of preeclampsia before 37 weeks of gestation. (a), the ROC curve of different screening programs for predicting the risk of preeclampsia before 32 weeks of gestation; (b), the ROC curve of different screening programs for predicting the risk of preeclampsia before 34 weeks of gestation; (c), the ROC curve of different screening programs for pregnant women for predicting the risk of developing preeclampsia before 37 weeks; (d), a summary of the sensitivity of different screenings of preeclampsia (<32 weeks, <34 weeks, and <37 weeks sum of the false positive rates).

When the PAPP-A + PLGF + MAP + UTPI quadruple screening program was used and the overall false positive rate was 15%, the screening positive rate before 32 weeks of gestation, 34 weeks of gestation, and before 37 weeks of gestation were 1.16%, 3.31%, and 15.08%, respectively, and the detection rates could reach 90%, 80%, and 63.64%, respectively, see Figure 1(d). The AUC of the PAPP-A + MAP + PLGF + UTPI scheme was 0.975, which was the largest among all schemes. The AUC of the UTPI scheme is 0.758, which is the smallest among all schemes.

### 3.3. Multi-Index Combined Risk Assessment Is More Suitable for Predicting Early-Onset Preeclampsia

**Figure 2:**
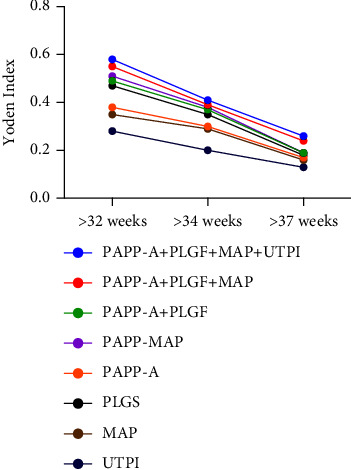
The changing trend of the Youden index of each screening program at different gestational weeks.

The ROC curve was used to analyze the sensitivity and specificity of each screening program in predicting the occurrence of preeclampsia before 32 weeks, 34 weeks, and 37 weeks of gestation, and the Youden index (sensitivity + specificity -1) of each coordinate point was calculated. The Youden index of each screening program for predicting preeclampsia showed a downward trend after 32 weeks of gestation, indicating that all screening programs had the greatest sensitivity and specificity for predicting early-onset preeclampsia, as shown in Figure 2.

## 4. Discussion

The incidence of preeclampsia is as high as 2% to 8% [4], and it seriously affects the safety of mothers and babies. More and more experts are aware of the importance of screening for preeclampsia [4–7]. The traditional screening program for preeclampsia is mainly based on maternal factors for risk assessment, including a previous history of gestational hypertension, chronic hypertension, chronic kidney disease, diabetes, or autoimmune diseases. However, in practice, most patients with preeclampsia do not present with the above factors. More and more evidence shows that the serological indicators PAPP-A and PLGF in the first trimester are related to the risk of preeclampsia, but the predictive value of a single indicator is not ideal [8–12]. Therefore, combinations of multiple indicators were used in this study to predict the risk of preeclampsia.

We found that before 37 weeks of gestation, the most effective screening regimen for preeclampsia was the PAPP-A + PLGF + MAP + UTPI quadruple regimen, which was similar to the previous study [13]. In addition, the UTPI prediction scheme alone was the worst among the screening schemes and was significantly different from other screening schemes. This may be related to the technical requirements of UTPI, which not all clinicians have mastered.

PAPP-A is a macromolecular glycoprotein secreted by placental trophoblast and decidual cells. PAPP-A levels decrease in pregnant women with preeclampsia due to placental dysfunction. PLGF promotes the maturation of the placental vasculature and is mainly expressed locally in the placenta. The level of PLGF affects placental vascular endothelial cells and trophoblast cells, and its decreased level induces preeclampsia. We found that after 32 weeks of gestation, with the increase of gestational weeks, the Youden index of the ROC curve of each screening program for preeclampsia showed a downward trend, which is similar to the conclusion of previous studies [14–16] that PAPP-A and PLGF are more closely related to the onset of early-onset preeclampsia.

Studies have shown [17] that the level of cell-free fetal RNA (cffRNA) in maternal peripheral blood is abnormal in the peripheral blood of pregnant women with preeclampsia. The placenta is the main source of cffRNA in maternal peripheral blood, and cffRNA is dependent on fetal sex. Given that cffRNA level directly reflects fetal gene expression pattern and can be noninvasively detected, cffRNA is considered as a potential biomarker for preeclampsia screening, but the time window of cffRNA detection is uncertain. For the reliability of preeclampsia screening, distinguishing gestational hypertension from preeclampsia, especially early-onset preeclampsia, is still needed. Other factors should also be considered when predicting preeclampsia [18–24].

In conclusion, we found that the optimal program for preeclampsia screening in pregnant women before 37 weeks of gestation is PAPP-A + PLGF + MAP + UTPI, which is extremely valuable in the prediction of early-onset preeclampsia.

## Figures and Tables

**Table 1:** Basic information of pregnant women participating in preeclampsia screening.

	Control group (*n* = 6116)	Preeclampsia group (*n* = 22)	*F/Z*	*P*
Expected age (years)^#^	28.10 ± 3.54	27.85 ± 3.72	0.11	0.74
weight (kg)^#^	56.93 ± 9.21	60.27 ± 9.18	2.88	0.90
height (cm)^#^	160.69 ± 4.96	160.18 ± 4.85	0.23	0.63
MAP^#^	1.01 ± 0.11	1.14 ± 0.12	32.69^*∗*^	<0.01
PAPP-A^#^	1.13 ± 0.69	0.68 ± 0.36	9.34^*∗*^	<0.01
PLGF^$^	0.77 (0.59∼1.02)	0.38 (0.30∼0.55)	−5.01^*∗*^	<0.01
UTPI^$^	0.80 (0.67∼0.96)	0.92 (0.73∼1.10)	−2.37^*∗*^	0.02

^#^: Indicates that the normal distribution is obtained by the KS normality test, and the F test is used; ^$^: indicates that the nonnormal distribution is obtained by the KS normality test, and the Mann–Whitney test is used; ^*∗*^: indicates that the result was significantly different.

## Data Availability

Data will be made available upon request to the corresponding author.
